# Correlation Between Homogeneity of Different Composite Resins and Their Adhesion to Glass Fiber Posts: In Vitro Assessment

**DOI:** 10.3390/dj13070290

**Published:** 2025-06-27

**Authors:** Živilė Oleinikaitė, Gediminas Skirbutis, Greta Rutkauskaitė

**Affiliations:** 1 Faculty of Odontology, Medical Academy, Lithuanian University of Health Sciences, Eiveniu 2, LT-50161 Kaunas, Lithuania; 2 Department of Prosthodontics, Faculty of Odontology, Lithuanian University of Health Sciences, Sukileliu Ave. 51, LT-50106 Kaunas, Lithuania; gediminas.skirbutis@lsmuni.lt (G.S.); greta.rutkauskaite@lsmu.lt (G.R.)

**Keywords:** composite resin, glass fiber post, adhesion, homogeneity

## Abstract

**Background and Objectives:** This in vitro trial aimed to investigate if there is a correlation between the homogeneities of different composite materials and their adhesion to glass fiber posts (GFPs). **Materials and Methods:** Twenty intact human upper jaw central incisors extracted due to periodontal diseases were selected for this trial. Endodontic treatment was performed according to ISO recommendations. A total of 4 mm of guttapercha was left in the apical region. Canals were prepared for post insertion. Teeth were randomly allocated into the two following groups depending on the core restorative material (*n* = 10): I—cores build up with light cured composite; II—cores build up with dual cured composite resin. GFPs were inserted and cores were rebuilt with different composite resins. Longitudinal cuts were made across the axis of the teeth and examined under a scanning electron microscope (SEM). Statistical analysis was accomplished using Mann–Whitney U and Spearman tests (*p* < 0.05). **Results:** In the group where the number and size of pores at the interface of GFPs were analyzed, pores were found only in the specimens restored with the light-cured “bulk-filled” composite. In the group where the number and size of pores in the core material were analyzed, pores were found in specimens restored with both the light-cured “bulk-filled” composite and dual-cured resin composite. However, the dual-cured resin composite yielded better results in terms of core integration. **Conclusions:** There is no statistically significant correlation between the homogeneities of different composite materials and their adhesion to GFP.

## 1. Introduction

Root canal therapy is one of the most often performed dental procedures, which prevents extractions and maintains dentition [1,2]. Indications for root canal therapy are irreversible pulpitis, pulp necrosis after dental trauma, excessive carious lesions, and apical periodontitis [1]. Root canal treatment is performed mechanically and chemically, during which microorganisms are removed and their re-entry into the canals is no longer possible [3]. The success rate of nonvital teeth is 68–97% [4]. However, moisture loss in dentin due to endodontic treatment leads to reduced resilience and an increased likelihood of fracture [5].

The condition of the periodontium, the load received during function, and the appropriate restoration have equal influence on the longevity of endodontically treated teeth as a successful root canal treatment [6]. Excessively damaged teeth often require intracanal restoration [7]. These days, glass fiber posts (GFPs) are one of the most chosen intracanal restorations because of their qualities. GFPs have high fracture resistance, an elasticity modulus similar to dentin, and favorable esthetic qualities [8,9].

These properties reduce the number of catastrophic complications and ameliorate the post-treatment survival outcomes of nonvital teeth [10].

After glass fiber post cementation, a core build up is needed. Composite resins are the main materials for core restoration. They reduce chair time and expenses for dental practitioners and patients, presenting good mechanical properties that help to resist stresses which can be produced during function [11]. Moreover, composites provide stress distributions of forces and reduce the probability of tensile and compressive failures [12].

One of the widely used light-cured composite materials is a bulk-filled composite. Over time, the latter has been improved in curing depth, which has led to the ability to place larger increments of up to 5 mm [13]. Manufacturers claim that bulk-filled composites have a lower shrinkage coefficient, lower polymerization shrinkage stress, and higher transparency that allows one to light-cure deeper layers [14,15].

Another popular material for core build up is dual-cured resin composite. This “2in1” material provides an opportunity to perform post cementation processes and core build up in one step. Dual-cured materials have chemical initiators and photochemical initiators in their composition, which ensure the polymerization of deeper layers [12]. In addition, monoblock adhesion among composite materials, dentin, and posts can create a restoration that has higher fracture resistance [16]. However, curing larger increments can introduce unwanted pores around GFPs and in the composite material that can reduce the strength of restoration [17].

Although GFPs are known to have fewer catastrophic complications, failures such as post debonding, core fracture, and post fracture still occur [18]. Though these complications are fixable, they require additional time, and new GFPs may be needed. Therefore, when restoring the core, the homogeneity of the composite and the integrity around the post and composite are of significant importance for the durability of the final restoration. The presence of pores both around the GFP and in the material itself negatively affects the strength of the abutment and increases the risk of fracture of the restoration during function [19]. Although SEM has already been used in similar studies, it is often combined with microtensile bond strength, shear bond strength, and fatigue testing. One of the main limitations of these tests is that the applied forces do not fully replicate the distribution, direction, or dynamic nature of masticatory and biting forces in the oral environment.

This in vitro trial aimed to evaluate if there is a correlation between the homogeneity of different composite materials and their adhesion to GFPs. It tested a null hypothesis stating that there is no statistically significant correlation between the homogeneities of different composite materials and their adhesion to GFPs.

## 2. Materials and Methods

Permission to conduct the research was issued by the Lithuanian University of Health Sciences ethics committee, with ethical approval number BEC-OF-33 (approved on 14 October 2019).

Twenty intact human central maxillary incisors extracted due to periodontal diseases were selected for this trial. The teeth were selected to be as uniform as possible in terms of root length and crown size. They were stored in saline for up to 3 months. Following access cavity preparation, the working lengths were measured, with a mean value of 18.75 mm.

Endodontic treatment was performed mechanically and chemically. Canals were prepared with Protaper Universal rotary files *(Dentsply Maillefer, Ballaigues, Switzerland)* with up to a #50 diameter according to ISO recommendations. A 2.5% sodium hypochlorite solution *(Chloraxd, Cerkamed, Stalowa Wola, Poland)* was used for rinsing. Canals were obturated with main and auxiliary guttapercha points *(Dentsply Maillefer, Ballaigues, Switzerland*) and a resin-based sealer *(Adseal, Meta Biomed, Müllheim, Germany)* by a method of lateral condensation.

Peeso reamers *(Peeso Reamer, Mani, Utsunomiya, Japan) #1, #2, and #3* were used for guttapercha removal from the canals. A total of 4 mm of obturator material was left in the apical region, creating the post space with an average of 14.75 mm. A 1.4 diameter drill *(DentoClic Drills, Itena, Paris, France)* provided in a kit together with glass fiber posts was used to calibrate canals for post insertion. A chlorhexidine solution *(GLUCO-Chex, Cerkamed, Stalowa Wola, Poland)* was used for rinsing, and paper points were used to dry canals.

**Figure 1:**
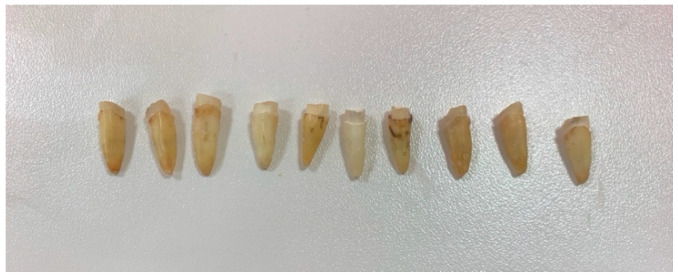
Shoulder margin and ferrule.

All teeth were decoronated, leaving a 2 mm ferrule and prepared with a 1 mm shoulder margin measured from the cementum–enamel junction (Figure 1).

Already silanized glass fiber posts *(DentoClic Translucent Glass fiber post, Itena, Paris, France)* with a diameter of 1.4 mm and a length of 18.5 mm were cut with a diamond bur using water for cooling according to the needed length. Canals were etched for 15 s with 36% phosphoric acid *(Phosphoric Acid Etching Gel, i-denta, Siauliai, Lithuania),* rinsed thoroughly with water for 15 s, dried using absorbent points, and were pretreated with ethanol.

Teeth were randomly allocated into the two following groups (*n* = 10) depending on the materials used for core build up (I, II):

I. Cores built up with a light-cured composite “bulk-filled” *(One Bulk Fill Restorative, 3M ESPE, St. Paul, MN, USA)*.
II. Cores built up with a dual-cured resin composite—dedicated for core build ups and GFP cementation *(Dentocore body, Itena, Paris, France)*.

### 2.1. Glass Fiber Post Cementation and Core Build up

**Figure 2:**
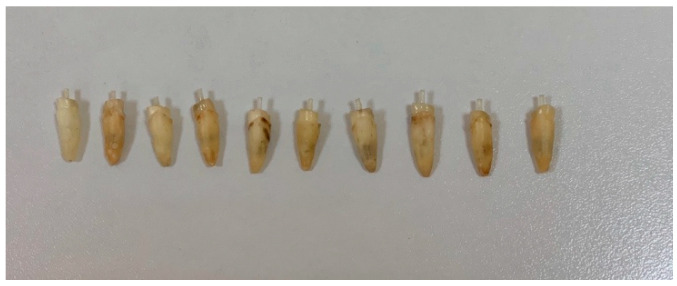
GFP cemented into root canals.

**Figure 3:**
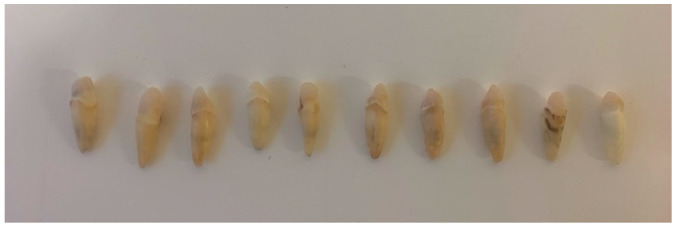
Prepared core shape for maxillary incisor crown.

Group I: A microbrush was used for adhesive (Single Bond Universal Adhesive, 3M ESPE, Neuss, Germany) application into the canals; the excess was removed with paper points and the adhesive was light-cured for 20 s. The adhesive was also applied on the GFP using the microbrush and was rubbed for 20 s; then, the adhesive was applied on the coronal dentin and light-cured for 20 s. Dual-cured resin composite was injected into the canals using an intracanal applicator. It was also applied on GFPs. The GFP was inserted into the canals and it was light-cured for 5 s. Any excess composite resin was removed in the elastic phase and light-cured for 15 s (Figure 2). The “bulk-filled’ composite was used to create an abutment of 5 mm height from the cementum–enamel junction in one increment and was light cured, respectively, for 20 s on each surface of the core. A handpiece was used to imitate the prepared maxillary incisors for dental crowns, trying to maintain a wall convergence of approximately 6° (Figure 3).

Group II: A microbrush was used for adhesive application into the canals, and the excess was removed with paper points, after which the adhesive was light-cured for 20 s. The adhesive was also applied to the GFP using the microbrush and rubbed for 20 s; then, the adhesive was applied on the coronal dentin and light-cured for 20 s. The dual-cured resin composite was injected into the canals using an intracanal applicator. It was also applied on the GFP. The GFP was inserted into the canals. Immediately, in order to create a monoblock adhesion, the same composite resin was used create an abutment of 5 mm height from the cementum–enamel junction and was light-cured for 40 s. The chemical polymerization of deeper layers was reached in 4.5 min. A handpiece was used to imitate the prepared maxillary incisors for dental crowns, trying to maintain a wall convergence of approximately 6° (Figure 3).

### 2.2. Sample Assessment

Longitudinal cuts were made across the axis of the teeth using diamond disks *(Diamond Bur, Dentaurum, Ispringen, Germany).* Sections were made 2 mm and 4 mm above the abutment, and samples of 2 mm width were created.

Sections were allowed to dry at room temperature for 24 h, polished using grit silicon carbide paper, and sputter-coated with gold before being observed under a scanning electron microscope (SEM) *(SEM Carl Zeiss EVO MA10, Cambridge, UK).*

Different magnifications (×50, ×100) were used for the observation of the integration of different composite materials with glass fiber posts, and the homogeneities of composite materials were evaluated according to the presence/absence, quantity, and size of the pores.

0 points—no pores;

1 point—one pore ≤ 200 µm;

2 points—pore size 200–500 µm;

3 points—pore size 500 µm–1 mm;

4 points—pore size > 1 mm.

An evaluation was made by two independent investigators. In the case of disagreement, the worse point was chosen.

### 2.3. Data Analysis

Statistical analysis was accomplished using SPSS version 22 on the gathered data. A *p* value < 0.05 was considered statistically significant. The nonparametric Mann–Whitney U tests were used to compare the pore scores between Group I and Group II for interface and internal porosity measures. Spearman’s rank correlation was used to assess the relationship between the interface pore score and the internal pore score across all samples.

## 3. Results

**Figure 4:**
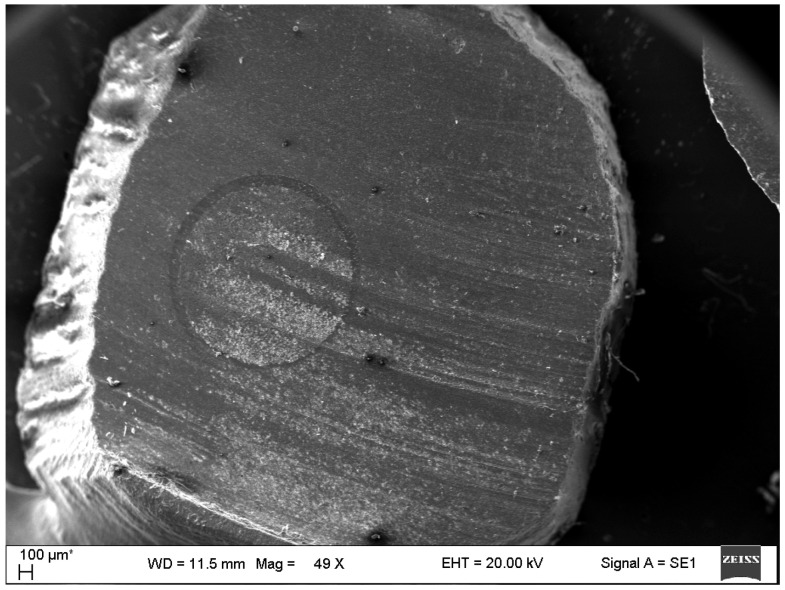
Dual-cured resin composite’s integration with GFP (score 0).

**Figure 5:**
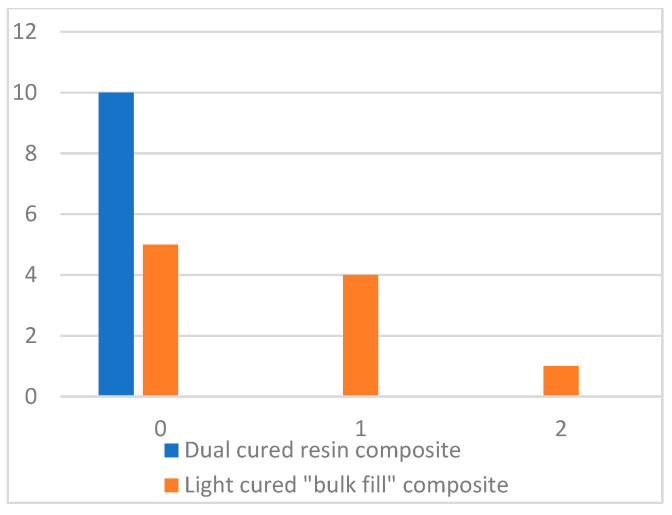
Different composite materials’ integration with GFP expressed as pore distribution along GFP.

The SEM examination of samples around the GFP revealed pores only in cores rebuilt with the light-cured “bulk-filled” composite. The dual-cured resin composite showed complete integration with the GFP (Figure 4). Half of the cores rebuilt with the light-cured composite also showed complete integration. In the group of cores rebuilt with the light-cured composite, one small pore (<200 µm) was found in each of the four cores and one pore (<500 µm) was found in one core (Figure 5).

The Mann–Whitney U test showed that the results between the groups were statistically significant (*p* < 0.05) (Table 1).

Accordingly, the size and quantity of pores in the light-cured “bulk-filled” composite are statistically significantly greater than in the dual-cure composite resin.

**Figure 6:**
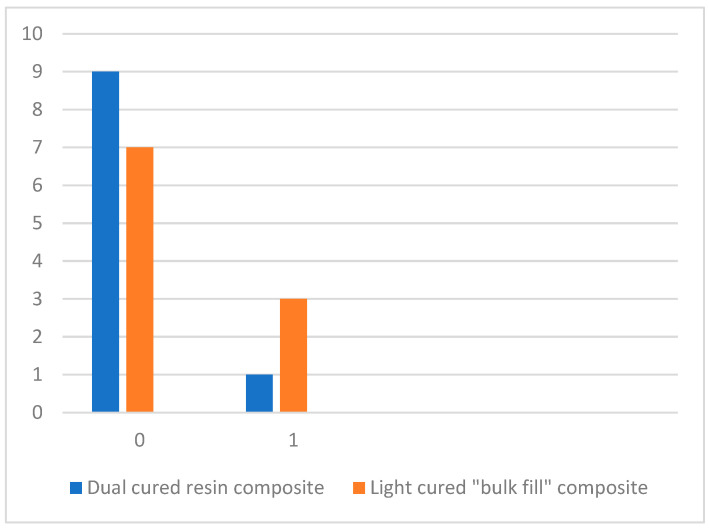
Homogeneity of different composite materials expressed as pore distribution.

Pores were found not only around the GFP but also in the core material itself. Pores were detected in both groups. The homogeneity of the dual-cured resin composite was better than that of the light-cured composite. Nine cores rebuilt with the dual-cured resin composite appeared to have no pores at all (score 0) and one core exhibited a single pore < 200 µm. Therefore, the light-cured “bulk-filled” composite exhibited greater porosity. One small pore (<200 µm) was found in each of three cores rebuilt with the light-cured composite (Figure 6).

The Mann–Whitney U test showed that the results between the groups were not statistically significant (*p* > 0.05) (Table 2).

Spearman’s test was performed to assess if there was a correlation between the presence of pores around the GFP and the presence of pores in the composite material. The test showed correlation (0.330), but it was not statistically significant (*p* = 0.156) (Table 3).

## 4. Discussion

A correlation was found between the integration of different composite resins with a GFP and the homogeneity of different composite resins, but it was not statistically significant (*p* > 0.05); thus, the null hypothesis was accepted.

Up to now, the conducted research is one of a few studies that have investigated the integration of different composite materials with a GFP and the homogeneities of core abutments using the scanning electron microscopy method to evaluate the absence, presence, and size of pores; however, none investigate the correlation.

The homogeneities of the composite, post, and core unit are of great importance, because they play an important role in the longevity of the final restoration [20]. Most prevalent complications of GFPs are debonding, post fracture, core debonding, and core fracture, which are related to adhesive bonding among the GFP, composite resin, and root canal dentin [21,22,23]. Gap formation along the GFP and in the core material has an influence on the strength of the abutment and increases the risk of fracture [24].

In this in vitro trial, the homogeneities of a dual-cured resin composite and light-cured “bulk-filled” composite were compared. The dual-cured resin composite showed better results (not statistically significant (*p* = 0.301)). It is known that one of the light-cured “bulk-filled” composites’ advantages is that larger increments of up to 5 mm can be polymerized at once, preventing gap formation along smaller increments [13]; however, while “bulk-filled” composites are formulated to have lower shrinkage stress, curing large increments at once can still generate internal stress, potentially leading to gaps and microleakage [25]. Dual-cured resin composites come in an automix delivery system and are technique-sensitive; therefore, proper mixing and placement are important to ensure an even cure. If not mixed correctly, the chemical cure component may not activate properly and voids may appear [26]. Filler load also has a significant impact on void formation. The light-cured “bulk-filled” composite has higher filler content than the dual-cured resin composite [27], though this fact is adverse to results in this study. The authors would assume that in this in vitro trial, the dual-cured resin composite showed better integration than the light-cured “bulk-filled” composite as the dual-cured resin is made of a more flowable material than the light-cured “bulk-filled” composite.

In 2004, Monticelli et al. [20] evaluated the adhesion of a GFP to different composite materials and the integrity of different composite core materials, using both plastic shells *(RTD dental*) and no shells. The selected evaluation method was scanning electron microscopy, the same as in this in vitro trial [20]. Three hybrid light-cured composites, *Z100 (3M-ESPE)*, *Lumiglass (RTD)*, and *Gradia (GC)*, and one dual-cured resin composite, *Build-it! (Pentron)*, were chosen for this trial. In the group comparing the adhesion of composites to the GFP without shells, the light-cured composite *Z100* showed significantly better results, which is different from the results in our trial. The group with shell’s best core integrity was reached with the dual-composite material *Build-it!* The latter material showed the best results when comparing the homogeneities of different composite resins used with a plastic matrix, and *Gradia* showed significantly better results in the group comparing the homogeneities of composite materials without shells. Altogether, the dual-cured resin composite *Build-it!* showed the best results in adhesion to a GFP and the homogeneity of a composite core used with a plastic matrix.

This study has several limitations that need to be considered. A larger number of samples could have determined different or statistically significant results while investigating correlation. On the other hand, both materials—the light-cured “bulk-filled” composite and the dual-cured resin composite demonstrated favorable outcomes in both adaptation and adhesion, and a broader number of samples could confirm that. Furthermore, a broader sample range would have enabled the use of plastic matrixes and a comparison of more composite resins from different manufacturers. Future studies should incorporate a pilot-based power analysis to guide sample size determination. Another limitation of this study is the technique used for material application on the GFP. The teeth were embedded in silicone molds to ensure stabilization during the procedure; however, this setup may have altered their positioning compared to their natural orientation in the oral cavity, potentially affecting the material application dynamics and clinical relevance.

## 5. Conclusions

Scanning electron microscopy of the samples revealed the presence of pores at the core material interface with the glass fiber post (GFP) only in the cores reconstructed with a light-cured bulk-filled composite. No pores were observed at the interface in the cores reconstructed with a dual-cured resin composite. However, within the core material itself, pores were found in both groups—those rebuilt with the light-cured “bulk-filled” composite and those with the dual-cured resin composite. There is no statistically significant correlation between the homogeneities of different composite materials and adhesion to GFPs. To ensure optimal material integration with the GFP and facilitate easier handling, the use of plastic matrixes is recommended when restoring the core with a dual-cured resin composite.

## Figures and Tables

**Table 1:** Statistical analysis of pores in the interface with the GFP between groups.

Groups	Average Rank	Z	*p*	Mann–Whitney U Test
Dual-cured resin composite	8.00	−2.500	0.012	25.000
Light-cured “bulk-filled” composite	12.00			

**Table 2:** Statistical analysis of pores in core material between groups.

Groups	Average Rank	Z	*p*	Mann–Whitney U Test
Dual-cured resin composite	9.50	−0.756	0.301	40.000
Light-cured “bulk-filled” composite	11.50			

**Table 3:** Correlation between the interface pore score and the internal pore score.

			Pores in Composite Material	Pores Around GFP
Spearman test	Pores in composite material	Correlation Coefficient	1.000	0.330
		Sig. (2-tailed)		0.156
		N	20	20
	Pores around GFP	Correlation Coefficient	0.330	1.000
		Sig. (2-tailed)	0.156	
		N	20	20

## Data Availability

Data are contained within the article.

## Abbreviations

The following abbreviations are used in this manuscript:

GFP	Glass fiber post
SEM	Scanning electron microscope
